# Supplementary material for: Sensor-supported measurement of adaptability of dogs (Canis familiaris) to a shelter environment: Nocturnal activity and behavior
Source: PLoS One. 2023 Jun 15;18(6):e0286429. doi: 10.1371/journal.pone.0286429 (PMC10270336; doi:10.1371/journal.pone.0286429)
Supplement: S1 Table — Estimated breed group [31], age class (in years, [13]), sex (female = f, male = m), neuter status (yes = y, no = n, unknown =?), reason for admission to the shelter (relinquished = R, stray = S, crisis boarding = CB), body weight class (in kg), body condition score (BCS) at intake in shelter (underweight BCS 1-3/ideal weight BCS 4-5/overweight BCS 6–9), food intake label (low eater or medium/good eater during the first two weeks in the shelter) and nocturnal video observations available (yes = y, no = n). (DOCX) [file pone.0286429.s001.docx]

**S1 Table. Demographics per shelter dog (SD) and control pet dog (CPD).**

| **Breed group** | **SD/**  **CPD** | **Age class** | **Sex** | **Neut. status** | **Reason**  **adm.** | **Kennel history** | **Body**  **weight** | **BCS intake** | **Eating during first two weeks** | **Video** |
| --- | --- | --- | --- | --- | --- | --- | --- | --- | --- | --- |
| Ancient & spitz breeds | SD | 1-4 yrs | f | ? | R | ? | 10-20 kg | Overweight | Low | Y |
| Ancient & spitz breeds | SD | 1-4 yrs | f | ? | CB | ? | <10 kg | Ideal weight | Low | N |
| Ancient & spitz breeds | SD | 1-4 yrs | m | n | S | ? | <10 kg | Ideal weight | Low | N |
| Ancient & spitz breeds | SD | 1-4 yrs | m | n | S | ? | 10-20 kg | Underweight | Medium/good | Y |
| Ancient & spitz breeds | SD | 1-4 yrs | m | y | R | ? | 10-20 kg | Overweight | Medium/good | N |
| Ancient & spitz breeds | SD | 1-4 yrs | m | n | R | ? | <10 kg | Ideal weight | Medium/good | Y |
| Ancient & spitz breeds | SD | 1-4 yrs | m | n | R | ? | <10 kg | Underweight | Low | Y |
| Herding dogs | SD | 1-4 yrs | f | y | R | ? | >30 kg | Overweight | Low | Y |
| Herding dogs | SD | 1-4 yrs | f | y | R | n | >20-30 kg | Overweight | Medium/good | Y |
| Herding dogs | SD | 1-4 yrs | m | n | R | ? | 10-20 kg | Ideal weight | Low | Y |
| Mastiff-like dogs | SD | 1-4 yrs | f | n | R | ? | >20-30 kg | Underweight | Medium/good | Y |
| Mastiff-like dogs | SD | 1-4 yrs | f | y | R | y | <10 kg | Overweight | Low | N |
| Mastiff-like dogs | SD | 1-4 yrs | f | n | S | ? | >30 kg | Overweight | Medium/good | Y |
| Mastiff-like dogs | SD | 1-4 yrs | f | n | S | ? | >20-30 kg | Overweight | Medium/good | N |
| Mastiff-like dogs | SD | 1-4 yrs | f | y | CP | y | 10-20 kg | Overweight | Medium/good | Y |
| Mastiff-like dogs | SD | 1-4 yrs | f | n | R | n | >20-30 kg | Overweight | Medium/good | Y |
| Mastiff-like dogs | SD | 1-4 yrs | m | y | R | ? | >20-30 kg | Ideal weight | Medium/good | Y |
| Mastiff-like dogs | SD | 1-4 yrs | m | n | R | y | >20-30 kg | Ideal weight | Medium/good | Y |
| Mastiff-like dogs | SD | 1-4 yrs | m | n | S | ? | >30 kg | Ideal weight | Medium/good | Y |
| Mastiff-like dogs | SD | 1-4 yrs | m | n | S | ? | >30 kg | Overweight | Medium/good | Y |
| Mastiff-like dogs | SD | 1-4 yrs | m | n | R | ? | >20-30 kg | Ideal weight | Medium/good | Y |
| Mastiff-like dogs | SD | 1-4 yrs | m | n | R | ? | >30 kg | Ideal weight | Medium/good | Y |
| Mastiff-like dogs | SD | 1-4 yrs | m | y | R | ? | >30 kg | Overweight | Medium/good | N |
| Mastiff-like dogs | SD | 1-4 yrs | m | n | S | ? | 10-20 kg | Overweight | Medium/good | Y |
| Mastiff-like dogs | SD | 1-4 yrs | m | n | S | ? | >30 kg | Ideal weight | Medium/good | N |
| Mastiff-like dogs | SD | 5-8 yrs | f | n | S | ? | >20-30 kg | Overweight | Medium/good | N |
| Mastiff-like dogs | SD | 5-8 yrs | m | n | S | ? | >20-30 kg | Ideal weight | Medium/good | N |
| Mastiff-like dogs | SD | 5-8 yrs | m | n | R | n | >20-30 kg | Overweight | Medium/good | Y |
| Mastiff-like dogs | SD | 9-13 yrs | m | n | S | ? | 10-20 kg | Overweight | Medium/good | Y |
| Mastiff-like dogs | SD | 9-13 yrs | m | y | R | n | >30 kg | Ideal weight | Medium/good | N |
| Mixed (undefinable) | SD | ? | m | n | CB | ? | 10-20 kg | Overweight | Low | N |
| Mixed (undefinable) | SD | 5-8 yrs | f | y | R | ? | 10-20 kg | Ideal weight | Medium/good | Y |
| Mixed (undefinable) | SD | 5-8 yrs | f | y | R | ? | 10-20 kg | Overweight | Medium/good | Y |
| Mixed (undefinable) | SD | 5-8 yrs | m | y | R | n | >20-30 kg | Overweight | Medium/good | Y |
| Retrievers | SD | 1-4 yrs | f | ? | S | ? | >20-30 kg | Overweight | Medium/good | Y |
| Retrievers | SD | 1-4 yrs | f | n | S | ? | >30 kg | Ideal weight | Medium/good | Y |
| Retrievers | SD | 1-4 yrs | m | y | R | ? | >30 kg | Overweight | Medium/good | Y |
| Retrievers | SD | 1-4 yrs | m | n | R | y | <10 kg | Overweight | Medium/good | N |
| Retrievers | SD | 5-8 yrs | m | n | R | y | >30 kg | Overweight | Medium/good | N |
| Scent hounds | SD | 1-4 yrs | m | n | CP | ? | <10 kg | Ideal weight | Low | N |
| Scent hounds | SD | 9-13 yrs | m | y | R | y | <10 kg | Overweight | Medium/good | Y |
| Small terriers | SD | 1-4 yrs | f | n | S | ? | <10 kg | Ideal weight | Medium/good | N |
| Small terriers | SD | 1-4 yrs | f | n | R | n | 10-20 kg | Overweight | Medium/good | Y |
| Small terriers | SD | 1-4 yrs | m | n | S | ? | <10 kg | Ideal weight | Medium/good | N |
| Small terriers | SD | 1-4 yrs | m | y | R | n | <10 kg | Overweight | Medium/good | Y |
| Small terriers | SD | 1-4 yrs | m | y | R | ? | <10 kg | Overweight | Medium/good | Y |
| Small terriers | SD | 1-4 yrs | m | y | S | ? | <10 kg | Overweight | Medium/good | Y |
| Small terriers | SD | 5-8 yrs | f | n | R | n | 10-20 kg | Overweight | Low | Y |
| Small terriers | SD | 5-8 yrs | m | y | R | ? | <10 kg | Overweight | Medium/good | Y |
| Small terriers | SD | 9-13 yrs | m | n | R | y | <10 kg | Overweight | Medium/good | N |
| Spaniels | SD | 1-4 yrs | m | y | R | y | 10-20 kg | Overweight | Medium/good | Y |
| Toy dogs | SD | 1-4 yrs | f | y | R | ? | <10 kg | Overweight | Low | Y |
| Toy dogs | SD | 1-4 yrs | m | n | R | ? | <10 kg | Ideal weight | Medium/good | Y |
| Toy dogs | SD | 9-13 yrs | m | n | S | ? | <10 kg | Ideal weight | Low | N |
| Working dogs | SD | 1-4 yrs | f | n | CB | ? | <10 kg | Ideal weight | Low | Y |
| Ancient & spitz breeds | CPD | 1-4 yrs | f | y |  |  | <10 kg | Ideal weight |  |  |
| Ancient & spitz breeds | CPD | 1-4 yrs | f | n |  |  | 10-20 kg | Ideal weight |  |  |
| Ancient & spitz breeds | CPD | 1-4 yrs | m | y |  |  | <10 kg | Ideal weight |  |  |
| Ancient & spitz breeds | CPD | 9-13 yrs | m | n |  |  | >20-30 kg | Ideal weight |  |  |
| Herding dogs | CPD | 1-4 yrs | m | y |  |  | 10-20 kg | Underweight |  |  |
| Mastiff-like dogs | CPD | 1-4 yrs | f | y |  |  | >20-30 kg | Ideal weight |  |  |
| Mastiff-like dogs | CPD | 1-4 yrs | f | y |  |  | >20-30 kg | Ideal weight |  |  |
| Mastiff-like dogs | CPD | 1-4 yrs | m | y |  |  | >20-30 kg | Ideal weight |  |  |
| Mastiff-like dogs | CPD | 1-4 yrs | m | y |  |  | 10-20 kg | Ideal weight |  |  |
| Mastiff-like dogs | CPD | 5-8 yrs | m | y |  |  | >20-30 kg | Ideal weight |  |  |
| Mastiff-like dogs | CPD | 5-8 yrs | m | y |  |  | >20-30 kg | Overweight |  |  |
| Mastiff-like dogs | CPD | 5-8 yrs | m | y |  |  | >30 kg | Overweight |  |  |
| Mixed (undefinable) | CPD | 1-4 yrs | f | y |  |  | 10-20 kg | Ideal weight |  |  |
| Mixed (undefinable) | CPD | 1-4 yrs | m | y |  |  | 10-20 kg | Ideal weight |  |  |
| Retrievers | CPD | 1-4 yrs | f | y |  |  | >20-30 kg | Ideal weight |  |  |
| Retrievers | CPD | 5-8 yrs | f | y |  |  | >20-30 kg | Overweight |  |  |
| Scent hounds | CPD | 1-4 yrs | m | n |  |  | <10 kg | Ideal weight |  |  |
| Small terriers | CPD | 1-4 yrs | m | y |  |  | 10-20 kg | Ideal weight |  |  |
| Small terriers | CPD | 1-4 yrs | m | y |  |  | <10 kg | Ideal weight |  |  |
| Small terriers | CPD | 5-8 yrs | f | y |  |  | <10 kg | Ideal weight |  |  |
| Toy dogs | CPD | 1-4 yrs | m | y |  |  | <10 kg | Ideal weight |  |  |

Estimated breed group [31], age class (in years, [13]), sex (female = f, male = m), neuter status (yes = y, no = n, unknown = ?), reason for admission to the shelter (relinquished = R, stray = S, crisis boarding = CB), kennel history (yes = y, no = n, unknown = ?), body weight class (in kg), body condition score (BCS) at intake in shelter (underweight BCS 1-3/ideal weight BCS 4-5/overweight BCS 6-9), food intake label (low eater or medium/good eater during the first two weeks in the shelter) and nocturnal video observations available (yes = y, no = n).
