# Supplementary material for: Sensor-supported measurement of adaptability of dogs (Canis familiaris) to a shelter environment: Nocturnal activity and behavior
Source: PLoS One. 2023 Jun 15;18(6):e0286429. doi: 10.1371/journal.pone.0286429 (PMC10270336; doi:10.1371/journal.pone.0286429)
Supplement: S2 Table — Estimated parameter (EP) and 95% confidence intervals (CI) of VMCpm during the night (0:00–4:00 h) for night (after intake) and neuter status, that both significantly explained the VMCpm variability. Conditional F-testing revealed F, DF’s and significance of the different terms in the models. 1 Estimated mean on reference night and neuter status. 2 Estimated ratio of mean of specified night and mean on reference night. 3 Estimated ratio of mean of specified neuter status and mean in reference neuter status. (DOCX) [file pone.0286429.s002.docx]

**S2 Table.** **Full model results of nocturnal activity accelerometer outputs: Vector Magnitude Counts per minute (VMCpm) in the shelter dog group.**

|  |  | *Vector Magnitude Counts per minute* | | | | | |
| --- | --- | --- | --- | --- | --- | --- | --- |
|  |  | Estimated | | Conditional F-test | | | |
| **Category** |  | **EP** | **95% CI** | **F** | **NumDF** | **DenDF** | **Sign.** |
| Reference | Night 1, neutered | 194.77^1^ | 138.18-274.55 | 3797.76 | 1 | 511 | <.0001 |
| Night | Night 2 versus night 1 | 0.72^2^ | 0.49-1.06 | 7.42 | 12 | 511 | <.0001 |
|  | Night 3 versus night 1 | 0.47^2^ | 0.33-0.68 |  |  |  |  |
|  | Night 4 versus night 1 | 0.46^2^ | 0.32-0.66 |  |  |  |  |
|  | Night 5 versus night 1 | 0.50^2^ | 0.35-0.71 |  |  |  |  |
|  | Night 6 versus night 1 | 0.39^2^ | 0.28-0.57 |  |  |  |  |
|  | Night 7 versus night 1 | 0.38^2^ | 0.26-0.54 |  |  |  |  |
|  | Night 8 versus night 1 | 0.37^2^ | 0.26-0.53 |  |  |  |  |
|  | Night 9 versus night 1 | 0.37^2^ | 0.26-0.53 |  |  |  |  |
|  | Night 10 versus night 1 | 0.37^2^ | 0.26-0.53 |  |  |  |  |
|  | Night 11 versus night 1 | 0.37^2^ | 0.26-0.53 |  |  |  |  |
|  | Night 12 versus night 1 | 0.30^2^ | 0.21-0.42 |  |  |  |  |
|  | Night 13 versus night 1 | 0.29^2^ | 0.20-0.42 |  |  |  |  |
| Neuter status | Neutered versus not neutered | 1.12^3^ | 0.82-1.53 | 3.43 | 2 | 52 | 0.0399 |
|  | Unknown versus not neutered | 2.33^3^ | 1.21-4.48 |  |  |  |  |

Estimated parameter (EP) and 95% confidence intervals (CI) of *VMCpm* during the night (0:00-4:00 h) for night (after intake) and neuter status, that both significantly explained the *VMCpm* variability. Conditional F-testing revealed F, DF’s and significance of the different terms in the models.

^1^ Estimated mean on reference night and neuter status

^2^ Estimated ratio of mean of specified night and mean on reference night.

^3^ Estimated ratio of mean of specified neuter status and mean in reference neuter status.
