# Supplementary material for: Sensor-supported measurement of adaptability of dogs (Canis familiaris) to a shelter environment: Nocturnal activity and behavior
Source: PLoS One. 2023 Jun 15;18(6):e0286429. doi: 10.1371/journal.pone.0286429 (PMC10270336; doi:10.1371/journal.pone.0286429)
Supplement: S3 Table — Estimated parameter (EP) and 95% confidence intervals (CI) of % active during the night (0:00–4:00 h) for night (after intake) and neuter status, that both significantly explained the % active variability. Conditional F-testing revealed F, DF’s and significance of the different terms in the models. 1 Estimated mean on reference night and neuter status. 2 Estimated ratio of mean of specified night and mean on reference night. 3 Estimated ratio of mean of specified neuter status and mean of reference neuter status. (DOCX) [file pone.0286429.s003.docx]

**S3 Table.** **Full model results of nocturnal activity accelerometer outputs: Percentage of time spent active in the shelter dog group.**

|  |  | *% of time spent active* | | | | | |
| --- | --- | --- | --- | --- | --- | --- | --- |
|  |  | Estimated | | Conditional F-test | | | |
| **Category** |  | **EP** | **95% CI** | **F** | **NumDF** | **DenDF** | **Sign.** |
| Reference | Night 1, neutered | 20^1^ | 16-26 | 1704.17 | 1 | 511 | <.0001 |
| Night | Night 2 versus night 1 | 0.75^2^ | 0.58-0.98 | 8.07 | 12 | 511 | <.0001 |
|  | Night 3 versus night 1 | 0.57^2^ | 0.44-0.74 |  |  |  |  |
|  | Night 4 versus night 1 | 0.56^2^ | 0.43-0.72 |  |  |  |  |
|  | Night 5 versus night 1 | 0.57^2^ | 0.44-0.74 |  |  |  |  |
|  | Night 6 versus night 1 | 0.51^2^ | 0.39-0.66 |  |  |  |  |
|  | Night 7 versus night 1 | 0.46^2^ | 0.35-0.59 |  |  |  |  |
|  | Night 8 versus night 1 | 0.44^2^ | 0.34-0.57 |  |  |  |  |
|  | Night 9 versus night 1 | 0.46^2^ | 0.35-0.59 |  |  |  |  |
|  | Night 10 versus night 1 | 0.47^2^ | 0.36-0.61 |  |  |  |  |
|  | Night 11 versus night 1 | 0.43^2^ | 0.33-0.56 |  |  |  |  |
|  | Night 12 versus night 1 | 0.40^2^ | 0.31-0.51 |  |  |  |  |
|  | Night 13 versus night 1 | 0.36^2^ | 0.28-0.47 |  |  |  |  |
| Neuter status | Neutered versus not neutered | 1.02^3^ | 0.81-1.29 | 3.62 | 2 | 52 | 0.0338 |
|  | Unknown versus not neutered | 1.91^3^ | 1.18-3.11 |  |  |  |  |

Estimated parameter (EP) and 95% confidence intervals (CI) of *% active* during the night (0:00-4:00 h) for night (after intake) and neuter status, that both significantly explained the *% active* variability. Conditional F-testing revealed F, DF’s and significance of the different terms in the models.

^1^ Estimated mean on reference night and neuter status

^2^ Estimated ratio of mean of specified night and mean on reference night.

^3^ Estimated ratio of mean of specified neuter status and mean of reference neuter status.
