# Supplementary material for: Sensor-supported measurement of adaptability of dogs (Canis familiaris) to a shelter environment: Nocturnal activity and behavior
Source: PLoS One. 2023 Jun 15;18(6):e0286429. doi: 10.1371/journal.pone.0286429 (PMC10270336; doi:10.1371/journal.pone.0286429)
Supplement: S4 Table — Estimated parameter (EP) and 95% confidence intervals (CI) of # inactive during the night (0:00–4:00 h) for night (after intake) and other factors that significantly explained the # inactive variability. Conditional F-testing revealed F, DF’s and significance of the different terms in the models. 1 Estimated mean on reference night, weight class and kennel history. 2 Estimated ratio of mean of specified night and mean on reference night. 3 Estimated ratio of mean of specified weight class and mean of reference weight class. 4 Estimated ratio of mean of specified kennel history and mean of reference kennel history. (DOCX) [file pone.0286429.s004.docx]

**S4 Table.** **Full model results of nocturnal activity accelerometer outputs: Number of inactive bouts in the shelter dog group.**

|  |  | *# inactive* | | | | | |
| --- | --- | --- | --- | --- | --- | --- | --- |
|  |  | Estimated | | Conditional F-test | | | |
| **Category** |  | **EP** | **95% CI** | **F** | **NumDF** | **DenDF** | **Sign.** |
| Reference | Night 1, 10-20 kg, had kennel history | 39.29^1^ | 31.66-48.76 | 10168.64 | 1 | 511 | <.0001 |
| Night | Night 2 versus night 1 | 0.85^2^ | 0.74-0.98 | 5.40 | 12 | 511 | <.0001 |
|  | Night 3 versus night 1 | 0.75^2^ | 0.66-0.87 |  |  |  |  |
|  | Night 4 versus night 1 | 0.74^2^ | 0.65-0.85 |  |  |  |  |
|  | Night 5 versus night 1 | 0.73^2^ | 0.64-0.84 |  |  |  |  |
|  | Night 6 versus night 1 | 0.74^2^ | 0.64-0.85 |  |  |  |  |
|  | Night 7 versus night 1 | 0.70^2^ | 0.61-0.80 |  |  |  |  |
|  | Night 8 versus night 1 | 0.67^2^ | 0.58-0.77 |  |  |  |  |
|  | Night 9 versus night 1 | 0.70^2^ | 0.61-0.80 |  |  |  |  |
|  | Night 10 versus night 1 | 0.69^2^ | 0.60-0.79 |  |  |  |  |
|  | Night 11 versus night 1 | 0.71^2^ | 0.62-0.82 |  |  |  |  |
|  | Night 12 versus night 1 | 0.65^2^ | 0.57-0.74 |  |  |  |  |
|  | Night 13 versus night 1 | 0.65^2^ | 0.56-0.75 |  |  |  |  |
| Weight class | <10 kg versus 10-20 kg | 0.91^3^ | 0.76-1.10 | 5.34 | 3 | 49 | 0.0029 |
|  | >20-30 kg versus 10-20 kg | 0.76^3^ | 0.62-0.94 |  |  |  |  |
|  | >30 kg versus 10-20 kg | 0.68^3^ | 0.55-0.84 |  |  |  |  |
| Kennel history | No history versus had history | 1.32^4^ | 1.01-1.71 | 2.80 | 2 | 49 | 0.0706 |
|  | Unknown versus had history | 1.34^4^ | 1.10-1.63 |  |  |  |  |

Estimated parameter (EP) and 95% confidence intervals (CI) of *# inactive* during the night (0:00-4:00 h) for night (after intake) and other factors that significantly explained the *# inactive* variability. Conditional F-testing revealed F, DF’s and significance of the different terms in the models.

^1^ Estimated mean on reference night, weight class and kennel history.

^2^ Estimated ratio of mean of specified night and mean on reference night.

^3^ Estimated ratio of mean of specified weight class and mean of reference weight class.

^4^ Estimated ratio of mean of specified kennel history and mean of reference kennel history.
