# Supplementary material for: Sensor-supported measurement of adaptability of dogs (Canis familiaris) to a shelter environment: Nocturnal activity and behavior
Source: PLoS One. 2023 Jun 15;18(6):e0286429. doi: 10.1371/journal.pone.0286429 (PMC10270336; doi:10.1371/journal.pone.0286429)
Supplement: S5 Table — Estimated parameter (EP) and 95% confidence intervals (CI) of # inactive >15 min during the night (0:00–4:00 h) for night (after intake) and other factors that significantly explained the # inactive >15 min variability. Conditional F-testing revealed F, DF’s and significance of the different terms in the models. 1 Estimated mean on reference night and weight class. 2 Estimated difference between mean of specified night and mean on reference night. 3 Estimated difference between mean of specified weight class and mean of reference weight class. (DOCX) [file pone.0286429.s005.docx]

**S5 Table. Full model results of nocturnal activity accelerometer outputs: Number of inactive bouts >15 minutes in the shelter dog group.**

|  |  | *# inactive > 15 min* | | | | | |
| --- | --- | --- | --- | --- | --- | --- | --- |
|  |  | Estimated | | Conditional F-test | | | |
| **Category** |  | **EP** | **95% CI** | **F** | **NumDF** | **DenDF** | **Sign.** |
| Reference | Night 1, <10 kg | 2.94^1^ | 2.21-3.67 | 1314.10 | 1 | 511 | <.0001 |
| Night | Night 2 versus night 1 | 1.05^2^ | 0.30-1.80 | 7.79 | 12 | 511 | <.0001 |
|  | Night 3 versus night 1 | 1.88^2^ | 1.15-2.62 |  |  |  |  |
|  | Night 4 versus night 1 | 1.79^2^ | 1.06-2.53 |  |  |  |  |
|  | Night 5 versus night 1 | 2.18^2^ | 1.45-2.91 |  |  |  |  |
|  | Night 6 versus night 1 | 2.23^2^ | 1.49-2.97 |  |  |  |  |
|  | Night 7 versus night 1 | 2.16^2^ | 1.42-2.89 |  |  |  |  |
|  | Night 8 versus night 1 | 2.54^2^ | 1.80-3.29 |  |  |  |  |
|  | Night 9 versus night 1 | 2.34^2^ | 1.60-3.08 |  |  |  |  |
|  | Night 10 versus night 1 | 2.12^2^ | 1.37-2.86 |  |  |  |  |
|  | Night 11 versus night 1 | 2.44^2^ | 1.70-3.19 |  |  |  |  |
|  | Night 12 versus night 1 | 2.75^2^ | 2.02-3.47 |  |  |  |  |
|  | Night 13 versus night 1 | 2.87^2^ | 2.11-3.64 |  |  |  |  |
| Weight class | 10-20 kg versus <10 kg | 0.08^3^ | -0.69-0.86 | 2.60 | 3 | 51 | 0.0623 |
|  | >20-30 kg versus <10 kg | 0.78^3^ | -0.03-1.60 |  |  |  |  |
|  | >30 kg versus <10 kg | 0.96^3^ | 0.11-1.80 |  |  |  |  |

Estimated parameter (EP) and 95% confidence intervals (CI) of *# inactive >15 min* during the night (0:00-4:00 h) for night (after intake) and other factors that significantly explained the *# inactive >15 min* variability. Conditional F-testing revealed F, DF’s and significance of the different terms in the models.

^1^ Estimated mean on reference night and weight class.

^2^ Estimated difference between mean of specified night and mean on reference night.

^3^ Estimated difference between mean of specified weight class and mean of reference weight class.
