# Supplementary material for: Sensor-supported measurement of adaptability of dogs (Canis familiaris) to a shelter environment: Nocturnal activity and behavior
Source: PLoS One. 2023 Jun 15;18(6):e0286429. doi: 10.1371/journal.pone.0286429 (PMC10270336; doi:10.1371/journal.pone.0286429)
Supplement: S6 Table — Estimated parameter (EP) and 95% confidence intervals (CI) of UCCR for day (after intake) and other factors that significantly explained UCCR variability. Conditional F-testing revealed F, DF’s and significance of the different terms in the models. 1 Estimated mean on reference day, weight class and kennel history. 2 Estimated ratio of mean of specified day and mean on reference day. 3 Estimated ratio of mean of specified weight class and mean of reference weight class. 4 Estimated ratio of mean of specified kennel history and mean of reference kennel history. (DOCX) [file pone.0286429.s006.docx]

**S6 Table.** **Model results for urinary cortisol/creatinine ratio (UCCR) of the shelter dog group.**

|  | | *Urinary cortisol/creatinine ratio (UCCR)* | | | | | |
| --- | --- | --- | --- | --- | --- | --- | --- |
| **Category** | | Estimated | | Conditional F-test | | | |
|  |  | **EP** | **95% CI** | **F** | **NumDF** | **DenDF** | **Sign.** |
| Reference | Day 1, <10 kg, had kennel history | 4.66^1^ | 2.96-7.34 | 462.39 | 1 | 285 | <.0001 |
| Day | Day 2 versus day 1 | 0.99^2^ | 0.83-1.18 | 2.65 | 6 | 285 | 0.0164 |
|  | Day 3 versus day 1 | 0.90^2^ | 0.73-1.12 |  |  |  |  |
|  | Day 5 versus day 1 | 0.84^2^ | 0.68-1.04 |  |  |  |  |
|  | Day 7 versus day 1 | 0.86^2^ | 0.66-1.12 |  |  |  |  |
|  | Day 9 versus day 1 | 0.69^2^ | 0.54-0.88 |  |  |  |  |
|  | Day 12 versus day 1 | 0.69^2^ | 0.52-0.91 |  |  |  |  |
| Weight class | 10-20 kg versus <10 kg | 1.14^3^ | 0.75-1.73 | 2.57 | 3 | 49 | 0.0103 |
|  | >20-30 kg versus <10 kg | 0.58^3^ | 0.37-0.92 |  |  |  |  |
|  | >30 kg versus <10 kg | 0.62^3^ | 0.40-0.96 |  |  |  |  |
| Kennel history | No history versus had history | 2.35^4^ | 1.31-4.23 | 2.57 | 2 | 49 | 0.0869 |
|  | Unknown versus had history | 1.65^4^ | 1.07-2.56 |  |  |  |  |

Estimated parameter (EP) and 95% confidence intervals (CI) of *UCCR* for day (after intake) and other factors that significantly explained *UCCR* variability. Conditional F-testing revealed F, DF’s and significance of the different terms in the models.

^1^ Estimated mean on reference day, weight class and kennel history.

^2^ Estimated ratio of mean of specified day and mean on reference day.

^3^ Estimated ratio of mean of specified weight class and mean of reference weight class.

^4^ Estimated ratio of mean of specified kennel history and mean of reference kennel history.
