# Supplementary material for: Sensor-supported measurement of adaptability of dogs (Canis familiaris) to a shelter environment: Nocturnal activity and behavior
Source: PLoS One. 2023 Jun 15;18(6):e0286429. doi: 10.1371/journal.pone.0286429 (PMC10270336; doi:10.1371/journal.pone.0286429)
Supplement: S7 Table — Estimated parameter (EP) and 95% confidence intervals (CI) of % active behaviour during the night (0:00–4:00 h) for night (after intake) and other factors that significantly explained % active behaviour variability. Conditional F-testing revealed F, DF’s and significance of the different terms in the models. 1 Estimated mean on reference night, weight class, sex and kennel history. 2 Estimated ratio of mean of specified night and mean on reference night. 3 Estimated ratio of mean of specified weight class and mean of reference weight class. 4 Estimated ratio of mean of specified sex and mean of reference sex. 5 Estimated ratio of mean of specified kennel history and mean of reference kennel history. (DOCX) [file pone.0286429.s007.docx]

**S7 Table.** **Model results for nocturnal activity behaviour: Percentage of time showing active behaviour in the shelter dog group.**

|  | | *% active behaviour* | | | | | |
| --- | --- | --- | --- | --- | --- | --- | --- |
| **Category** | | Estimated | | Conditional F-test | | | |
|  |  | **EP** | **95% CI** | **F** | **NumDF** | **DenDF** | **Sign.** |
| Reference | Night 1, <10 kg, male, had kennel history | 10.34^1^ | 5.84-18.31 | 753.37 | 1 | 212 | <.0001 |
| Night | Night 2 versus night 1 | 0.70^2^ | 0.51-0.96 | 12.00 | 6 | 212 | <.0001 |
|  | Night 3 versus night 1 | 0.49^2^ | 0.35-0.69 |  |  |  |  |
|  | Night 5 versus night 1 | 0.48^2^ | 0.34-0.69 |  |  |  |  |
|  | Night 7 versus night 1 | 0.36^2^ | 0.25-0.51 |  |  |  |  |
|  | Night 9 versus night 1 | 0.31^2^ | 0.22-0.44 |  |  |  |  |
|  | Night 12 versus night 1 | 0.26^2^ | 0.18-0.37 |  |  |  |  |
| Weight class | 10-20 kg versus <10 kg | 1.19^3^ | 0.75-1.89 | 3.99 | 3 | 30 | 0.0167 |
|  | >20-30 kg versus <10 kg | 0.77^3^ | 0.47-1.25 |  |  |  |  |
|  | >30 kg versus <10 kg | 0.47^3^ | 0.28-0.77 |  |  |  |  |
| Sex | Female versus male | 1.43^4^ | 1.00-2.04 | 7.65 | 1 | 30 | 0.0096 |
| Kennel history | No history versus had history | 1.95^5^ | 1.06-3.61 | 3.99 | 2 | 30 | 0.0291 |
|  | Unknown versus had history | 2.03^5^ | 1.21-3.41 |  |  |  |  |

Estimated parameter (EP) and 95% confidence intervals (CI) of *% active behaviour* during the night (0:00-4:00 h) for night (after intake) and other factors that significantly explained *% active behaviour* variability. Conditional F-testing revealed F, DF’s and significance of the different terms in the models.

^1^ Estimated mean on reference night, weight class, sex and kennel history.

^2^ Estimated ratio of mean of specified night and mean on reference night.

^3^ Estimated ratio of mean of specified weight class and mean of reference weight class.

^4^ Estimated ratio of mean of specified sex and mean of reference sex.

^5^ Estimated ratio of mean of specified kennel history and mean of reference kennel history.
