# Supplementary material for: Sensor-supported measurement of adaptability of dogs (Canis familiaris) to a shelter environment: Nocturnal activity and behavior
Source: PLoS One. 2023 Jun 15;18(6):e0286429. doi: 10.1371/journal.pone.0286429 (PMC10270336; doi:10.1371/journal.pone.0286429)
Supplement: S8 Table — Estimated parameter (EP) and 95% confidence intervals (CI) of % recumbent head up during the night (0:00–4:00 h) for night (after intake) and other factors that significantly explained % recumbent head up variability. Conditional F-testing revealed F, DF’s and significance of the different terms in the models. 1 Estimated mean on reference night, weight class and kennel history. 2 Estimated ratio of mean of specified night and mean on reference night. 3 Estimated ratio of mean of specified weight class and mean of reference weight class. 4 Estimated ratio of mean of specified kennel history and mean of reference kennel history. (DOCX) [file pone.0286429.s008.docx]

**S8 Table.** **Model results for nocturnal activity behaviour: Percentage of time showing recumbent head up in the shelter dog group.**

|  | | *% recumbent head up* | | | | | |
| --- | --- | --- | --- | --- | --- | --- | --- |
| **Category** | | Estimated | | Conditional F-test | | | |
|  |  | **EP** | **95% CI** | **F** | **NumDF** | **DenDF** | **Sign.** |
| Reference | Night 1, <10 kg, had kennel history | 4.49^1^ | 2.66-7.58 | 474.66 | 1 | 212 | <.0001 |
| Day | Night 2 versus night 1 | 0.80^2^ | 0.63-1.01 | 10.80 | 6 | 212 | <.0001 |
|  | Night 3 versus night 1 | 0.63^2^ | 0.49-0.81 |  |  |  |  |
|  | Night 5 versus night 1 | 0.61^2^ | 0.47-0.79 |  |  |  |  |
|  | Night 7 versus night 1 | 0.46^2^ | 0.36-0.60 |  |  |  |  |
|  | Night 9 versus night 1 | 0.46^2^ | 0.35-0.59 |  |  |  |  |
|  | Night 12 versus night 1 | 0.39^2^ | 0.30-0.50 |  |  |  |  |
| Weight class | 10-20 kg versus <10 kg | 1.66^3^ | 1.10-2.52 | 4.09 | 3 | 31 | 0.0148 |
|  | >20-30 kg versus <10 kg | 0.88^3^ | 0.56-1.39 |  |  |  |  |
|  | >30 kg versus <10 kg | 0.67^3^ | 0.42-1.06 |  |  |  |  |
| Kennel history | No history versus had history | 1.97^4^ | 1.12-3.46 | 4.69 | 2 | 31 | 0.0166 |
|  | Unknown versus had history | 2.03^4^ | 1.25-3.28 |  |  |  |  |

Estimated parameter (EP) and 95% confidence intervals (CI) of *% recumbent head up* during the night (0:00-4:00 h) for night (after intake) and other factors that significantly explained *% recumbent head up* variability. Conditional F-testing revealed F, DF’s and significance of the different terms in the models.

^1^ Estimated mean on reference night, weight class and kennel history.

^2^ Estimated ratio of mean of specified night and mean on reference night.

^3^ Estimated ratio of mean of specified weight class and mean of reference weight class.

^4^ Estimated ratio of mean of specified kennel history and mean of reference kennel history.
