# Supplementary material for: Sensor-supported measurement of adaptability of dogs (Canis familiaris) to a shelter environment: Nocturnal activity and behavior
Source: PLoS One. 2023 Jun 15;18(6):e0286429. doi: 10.1371/journal.pone.0286429 (PMC10270336; doi:10.1371/journal.pone.0286429)
Supplement: S9 Table — Estimated parameter values (EP) and 95% confidence intervals (CI) of % stationary during the night (0:00–4:00 h) for night (after intake) and other factors that significantly explained % stationary variability. Conditional F-testing revealed F, DF’s and significance of factors in the model. 1 Estimated mean in reference night, weight class, age class and kennel history. 2 Estimated ratio of mean of specified night and mean on reference night. 3 Estimated ratio of mean of specified weight class and mean in reference weight class. 4 Estimated ratio of mean of specified age class and mean of reference age class. 5 Estimated ratio of mean of specified kennel history and mean of reference kennel history at the same night. (DOCX) [file pone.0286429.s009.docx]

**S9 Table.** **Model results for nocturnal activity behaviour: Percentage of time showing stationary behaviour in the shelter dog group.**

|  | | | *% stationary* | | | | | |
| --- | --- | --- | --- | --- | --- | --- | --- | --- |
| **Category** | | | Estimated | | Conditional F-test | | | |
|  |  |  | **EP** | **95% CI** | **F** | **NumDF** | **DenDF** | **Sign.** |
| Reference | Night 1, <10 kg, 1-4 years, had kennel history | | 3.12^1^ | 0.66-14.76 | 18.31 | 1 | 198 | <.0001 |
| Night | Night 2 versus night 1 | | 0.39^2^ | 0.09-1.73 | 9.75 | 6 | 198 | <.0001 |
|  | Night 3 versus night 1 | | 0.47^2^ | 0.09-2.41 |  |  |  |  |
|  | Night 5 versus night 1 | | 0.36^2^ | 0.07-1.93 |  |  |  |  |
|  | Night 7 versus night 1 | | 0.29^2^ | 0.05-1.53 |  |  |  |  |
|  | Night 9 versus night 1 | | 0.28^2^ | 0.05-1.50 |  |  |  |  |
|  | Night 12 versus night 1 | | 0.25^2^ | 0.05-1.35 |  |  |  |  |
| Weight class | 10-20 kg versus <10 kg | | 1.26^3^ | 0.58-2.70 | 2.28 | 3 | 31 | 0.0986 |
|  | >20-30 kg versus <10 kg | | 0.94^3^ | 0.41-2.15 |  |  |  |  |
|  | >30 kg versus <10 kg | | 0.32^3^ | 0.14-0.73 |  |  |  |  |
| Age class | 5-7 yrs versus 1-4 yrs | | 0.29^4^ | 0.13-0.65 | 5.32 | 2 | 31 | 0.0103 |
|  | 8-13 yrs versus 1-4 yrs | | 0.54^4^ | 0.12-2.52 |  |  |  |  |
| Kennel history * night | No history versus had history | Night 1 | 10.11^5^ | 1.63-62.74 | 1.76 | 14 | 198 | 0.0474 |
|  |  | Night 2 | 10.79^5^ | 1.74-66.97 |  |  |  |  |
|  |  | Night 3 | 3.51^5^ | 0.57-21.78 |  |  |  |  |
|  |  | Night 5 | 7.31^5^ | 1.18-45.38 |  |  |  |  |
|  |  | Night 7 | 0.84^5^ | 0.13-5.19 |  |  |  |  |
|  |  | Night 9 | 1.02^5^ | 0.16-6.33 |  |  |  |  |
|  |  | Night 12 | 1.17^5^ | 0.19-7.24 |  |  |  |  |
|  | Unknown versus had history | Night 1 | 2.19^5^ | 0.46-10.54 |  |  |  |  |
|  |  | Night 2 | 4.36^5^ | 0.91-20.84 |  |  |  |  |
|  |  | Night 3 | 1.76^5^ | 0.37-8.43 |  |  |  |  |
|  |  | Night 5 | 2.63^5^ | 0.55-12.59 |  |  |  |  |
|  |  | Night 7 | 3.33^5^ | 0.69-16.03 |  |  |  |  |
|  |  | Night 9 | 1.74^5^ | 0.36-8.33 |  |  |  |  |
|  |  | Night 12 | 1.25^5^ | 0.26-6.03 |  |  |  |  |

Estimated parameter values (EP) and 95% confidence intervals (CI) of *% stationary* during the night (0:00-4:00 h) for night (after intake) and other factors that significantly explained *% stationary* variability. Conditional F-testing revealed F, DF’s and significance of factors in the model.

^1^ Estimated mean in reference night, weight class, age class and kennel history.

^2^ Estimated ratio of mean of specified night and mean on reference night.

^3^ Estimated ratio of mean of specified weight class and mean in reference weight class.

^4^ Estimated ratio of mean of specified age class and mean of reference age class.

^5^ Estimated ratio of mean of specified kennel history and mean of reference kennel history at the same night.
