# Supplementary material for: Sensor-supported measurement of adaptability of dogs (Canis familiaris) to a shelter environment: Nocturnal activity and behavior
Source: PLoS One. 2023 Jun 15;18(6):e0286429. doi: 10.1371/journal.pone.0286429 (PMC10270336; doi:10.1371/journal.pone.0286429)
Supplement: S10 Table — Estimated parameter values (EP) and 95% confidence intervals (CI) of % of movement during the night (0:00–4:00 h) for night (after intake) and neuter status, that both significantly explained the % of movement variability. Conditional F-testing revealed F, DF’s and significance of factors in the model. 1 Estimated mean on reference night and neuter status. 2 Estimated ratio of mean of specified night and mean on reference night. 3 Estimated ratio of mean of specified neuter status and mean of reference neuter status. (DOCX) [file pone.0286429.s010.docx]

**S10 Table.** **Model results for nocturnal activity behaviour: Percentage of time showing movement behaviour in the shelter dog group.**

|  | | *% movement* | | | | | |
| --- | --- | --- | --- | --- | --- | --- | --- |
| **Category** | | Estimated | | Conditional F-test | | | |
|  |  | **EP** | **95% CI** | **F** | **NumDF** | **DenDF** | **Sign.** |
| Reference | Night 1, intact | 1.45^1^ | 0.83 - 2.54 | 22.57 | 1 | 189 | <.0001 |
| Night | Night 2 versus night 1 | 0.62^2^ | 0.33-1.14 | 8.24 | 6 | 189 | <.0001 |
|  | Night 3 versus night 1 | 0.31^2^ | 0.16-0.61 |  |  |  |  |
|  | Night 5 versus night 1 | 0.30^2^ | 0.16-0.59 |  |  |  |  |
|  | Night 7 versus night 1 | 0.20^2^ | 0.10-0.40 |  |  |  |  |
|  | Night 9 versus night 1 | 0.14^2^ | 0.07-0.26 |  |  |  |  |
|  | Night 12 versus night 1 | 0.15^2^ | 0.08-0.30 |  |  |  |  |
| Neuter status | Neutered versus intact | 1.25^3^ | 0.72-2.19 | 3.04 | 2 | 30 | 0.0628 |
|  | Unknown versus intact | 4.08^3^ | 1.26-13.20 |  |  |  |  |

Estimated parameter values (EP) and 95% confidence intervals (CI) of *% of movement* during the night (0:00-4:00 h) for night (after intake) and neuter status, that both significantly explained the *% of movement* variability. Conditional F-testing revealed F, DF’s and significance of factors in the model.

^1^ Estimated mean on reference night and neuter status.

^2^ Estimated ratio of mean of specified night and mean on reference night.

^3^ Estimated ratio of mean of specified neuter status and mean of reference neuter status.
