# Supplementary material for: Sensor-supported measurement of adaptability of dogs (Canis familiaris) to a shelter environment: Nocturnal activity and behavior
Source: PLoS One. 2023 Jun 15;18(6):e0286429. doi: 10.1371/journal.pone.0286429 (PMC10270336; doi:10.1371/journal.pone.0286429)
Supplement: S11 Table — Estimated parameter values (EP) and 95% confidence intervals (CI) of transition in movements (rpm) during the night (0:00–4:00 h) for night (after intake) and age class, that both significantly explained the transitions in movement (rpm) variability. Conditional F-testing revealed F, DF’s and significance of factors in the model.1 Estimated mean on reference night and age class. 2 Estimated ratio of mean of specified night and mean on reference night. 3 Estimated ratio of mean of specified age class and mean of reference age class. (DOCX) [file pone.0286429.s011.docx]

**S11 Table.** **Model results for nocturnal activity behaviour: Rate per minute (RPM) of transitions in the different activity behaviours in the shelter dog group.**

|  | | *RPM of transitions in the different activity behaviours* | | | | | |
| --- | --- | --- | --- | --- | --- | --- | --- |
| **Category** | | Estimated | | Conditional F-test | | | |
|  |  | **EP** | **95% CI** | **F** | **NumDF** | **DenDF** | **Sign.** |
| Reference | Night 1, 1-4 yrs | 1.77^1^ | 1.38 - 2.28 | 13.06 | 1 | 212 | 0.0004 |
| Night | Night 2 versus night 1 | 0.97^2^ | 0.76-1.25 | 2.61 | 6 | 212 | 0.0186 |
|  | Night 3 versus night 1 | 0.72^2^ | 0.55-0.96 |  |  |  |  |
|  | Night 5 versus night 1 | 0.84^2^ | 0.63-1.13 |  |  |  |  |
|  | Night 7 versus night 1 | 0.77^2^ | 0.58-1.04 |  |  |  |  |
|  | Night 9 versus night 1 | 0.62^2^ | 0.46-0.83 |  |  |  |  |
|  | Night 12 versus night 1 | 0.73^2^ | 0.54-0.98 |  |  |  |  |
| Age class | 5-7 yrs versus 1-4 yrs | 0.63^3^ | 0.41-0.95 | 2.59 | 2 | 34 | 0.0900 |
|  | 8-13 yrs versus 1-4 yrs | 0.94^3^ | 0.48-1.87 |  |  |  |  |

Estimated parameter values (EP) and 95% confidence intervals (CI) of *transition in movements (rpm)* during the night (0:00-4:00 h) for night (after intake) and age class, that both significantly explained the *transitions in movement (rpm)* variability. Conditional F-testing revealed F, DF’s and significance of factors in the model.
^1^ Estimated mean on reference night and age class.

^2^ Estimated ratio of mean of specified night and mean on reference night.

^3^ Estimated ratio of mean of specified age class and mean of reference age class.
