# Supplementary material for: Sensor-supported measurement of adaptability of dogs (Canis familiaris) to a shelter environment: Nocturnal activity and behavior
Source: PLoS One. 2023 Jun 15;18(6):e0286429. doi: 10.1371/journal.pone.0286429 (PMC10270336; doi:10.1371/journal.pone.0286429)
Supplement: S12 Table — Estimated parameter values (EP) and 95% confidence intervals (CI) of oral behaviour (RPM) when the dog was in sight of the camera and active, during the night (0:00–4:00 h) for night (after intake). Conditional F-testing revealed F, DF’s and significance of factors in the model. 1 Estimated mean on reference night. 2 Estimated ratio of mean of specified night and mean on reference night. (DOCX) [file pone.0286429.s012.docx]

**S12 Table.** **Model results for nocturnal behavioural indicators of stress: Rate per minute (RPM) of oral behaviour (yawning + lip/snout licking) in the shelter dog group.**

|  | | *RPM of oral behaviour* | | | | | |
| --- | --- | --- | --- | --- | --- | --- | --- |
| **Category** | | Estimated | | Conditional F-test | | | |
|  |  | **EP** | **95% CI** | **F** | **NumDF** | **DenDF** | **Sign.** |
| Reference | Night 1 | 0.75^1^ | 0.50-1.12 | 2.41 | 1 | 212 | 0.1224 |
| Night | Night 2 versus night 1 | 0.97^2^ | 0.66-1.43 | 0.49 | 6 | 212 | 0.8131 |
|  | Night 3 versus night 1 | 0.84^2^ | 0.45-1.54 |  |  |  |  |
|  | Night 5 versus night 1 | 1.17^2^ | 0.82-1.65 |  |  |  |  |
|  | Night 7 versus night 1 | 0.97^2^ | 0.54-1.76 |  |  |  |  |
|  | Night 9 versus night 1 | 0.82^2^ | 0.44-1.55 |  |  |  |  |
|  | Night 12 versus night 1 | 1.13^2^ | 0.67-1.90 |  |  |  |  |

Estimated parameter values (EP) and 95% confidence intervals (CI) of *oral behaviour (RPM)* when the dog was in sight of the camera and active, during the night (0:00-4:00 h) for night (after intake). Conditional F-testing revealed F, DF’s and significance of factors in the model.
^1^ Estimated mean on reference night.

^2^ Estimated ratio of mean of specified night and mean on reference night.
