# Supplementary material for: Sensor-supported measurement of adaptability of dogs (Canis familiaris) to a shelter environment: Nocturnal activity and behavior
Source: PLoS One. 2023 Jun 15;18(6):e0286429. doi: 10.1371/journal.pone.0286429 (PMC10270336; doi:10.1371/journal.pone.0286429)
Supplement: S13 Table — Estimated parameter values (EP) and 95% confidence intervals (CI) of autogrooming (proportion of time) when the dog was in sight of the camera and active, during the night (0:00–4:00 h) for night (after intake) and other factors that significantly explained autogrooming variability. Conditional F-testing revealed F, DF’s and significance of factors in the model. 1 Estimated mean in reference night, weight class and age class. 2 Estimated ratio of mean of specified night and mean on reference night. 3 Estimated ratio of mean of specified weight class and mean in reference weight class at the same night. 4 Estimated ratio of mean of specified age class and mean of reference age class at the same night. (DOCX) [file pone.0286429.s013.docx]

**S13 Table.** **Model results for nocturnal behavioural indicators of stress: Proportion of time spent autogrooming in the shelter dog group.**

|  | | | *Proportion autogrooming* | | | | | |
| --- | --- | --- | --- | --- | --- | --- | --- | --- |
| **Category** | | | Estimated | | Conditional F-test | | | |
|  |  |  | **EP** | **95% CI** | **F** | **NumDF** | **DenDF** | **Sign.** |
| Reference | Night 1, <10 kg, 1-4 years | | 0.02^1^ | 0.01-0.05 | 277.58 | 1 | 177 | <.0001 |
| Night | Night 2 versus night 1 | | 1.77^2^ | 0.82-3.82 | 7.83 | 6 | 177 | <.0001 |
|  | Night 3 versus night 1 | | 7.09^2^ | 3.12-16.10 |  |  |  |  |
|  | Night 5 versus night 1 | | 3.68^2^ | 1.56-8.68 |  |  |  |  |
|  | Night 7 versus night 1 | | 3.99^2^ | 1.64-9.74 |  |  |  |  |
|  | Night 9 versus night 1 | | 4.01^2^ | 1.60-10.08 |  |  |  |  |
|  | Night 12 versus night 1 | | 5.21^2^ | 2.02-13.44 |  |  |  |  |
| Weight class * night | 10-20 kg versus <10 kg | Night 1 | 1.08^3^ | 0.44-2.65 | 1.59 | 21 | 177 | 0.0549 |
|  |  | Night 2 | 1.53^3^ | 0.57-4.13 |  |  |  |  |
|  |  | Night 3 | 0.58^3^ | 0.20-1.67 |  |  |  |  |
|  |  | Night 5 | 0.73^3^ | 0.24-2.24 |  |  |  |  |
|  |  | Night 7 | 1.10^3^ | 0.34-3.53 |  |  |  |  |
|  |  | Night 9 | 0.65^3^ | 0.20-2.17 |  |  |  |  |
|  |  | Night 12 | 0.93^3^ | 0.27-3.22 |  |  |  |  |
|  | >20-30 kg versus <10 kg | Night 1 | 5.08^3^ | 1.93-13.33 |  |  |  |  |
|  |  | Night 2 | 2.60^3^ | 0.91-7.39 |  |  |  |  |
|  |  | Night 3 | 0.63^3^ | 0.21-1.92 |  |  |  |  |
|  |  | Night 5 | 2.60^3^ | 0.80-8.44 |  |  |  |  |
|  |  | Night 7 | 0.80^3^ | 0.24-2.73 |  |  |  |  |
|  |  | Night 9 | 1.72^3^ | 0.49-6.12 |  |  |  |  |
|  |  | Night 12 | 0.31^3^ | 0.08-1.17 |  |  |  |  |
|  | >30 kg versus <10 kg | Night 1 | 3.50^3^ | 1.28-9.62 |  |  |  |  |
|  |  | Night 2 | 3.57^3^ | 1.17-10.96 |  |  |  |  |
|  |  | Night 3 | 1.02^3^ | 0.31-3.38 |  |  |  |  |
|  |  | Night 5 | 1.95^3^ | 0.55-6.89 |  |  |  |  |
|  |  | Night 7 | 0.84^3^ | 0.20-3.48 |  |  |  |  |
|  |  | Night 9 | 1.91^3^ | 0.49-7.43 |  |  |  |  |
|  |  | Night 12 | 0.83^3^ | 0.21-3.37 |  |  |  |  |
| Age class * night | 5-7 yrs versus 1-4 yrs | Night 1 | 0.57^4^ | 0.22-1.47 | 2.69 | 14 | 177 | 0.0013 |
|  |  | Night 2 | 1.48^4^ | 0.52-4.21 |  |  |  |  |
|  |  | Night 3 | 2.32^4^ | 0.76-7.11 |  |  |  |  |
|  |  | Night 5 | 2.03^4^ | 0.63-6.59 |  |  |  |  |
|  |  | Night 7 | 1.43^4^ | 0.42-4.87 |  |  |  |  |
|  |  | Night 9 | 1.33^4^ | 0.38-4.74 |  |  |  |  |
|  |  | Night 12 | 1.67^4^ | 0.45-6.20 |  |  |  |  |
|  | 8-13 yrs versus 1-4 yrs | Night 1 | 5.00^4^ | 1.10-22.64 |  |  |  |  |
|  |  | Night 2 | 1.08^4^ | 0.20-5.74 |  |  |  |  |
|  |  | Night 3 | 1.33^4^ | 0.22-7.96 |  |  |  |  |
|  |  | Night 5 | 5.42^4^ | 0.82-35.71 |  |  |  |  |
|  |  | Night 7 | 0.11^4^ | 0.02-0.81 |  |  |  |  |
|  |  | Night 9 | 0.34^4^ | 0.05-2.62 |  |  |  |  |
|  |  | Night 12 | 0.09^4^ | 0.01-0.69 |  |  |  |  |

Estimated parameter values (EP) and 95% confidence intervals (CI) of *autogrooming (proportion of time)* when the dog was in sight of the camera and active, during the night (0:00-4:00 h) for night (after intake) and other factors that significantly explained *autogrooming* variability. Conditional F-testing revealed F, DF’s and significance of factors in the model.

^1^ Estimated mean in reference night, weight class and age class.

^2^ Estimated ratio of mean of specified night and mean on reference night.

^3^ Estimated ratio of mean of specified weight class and mean in reference weight class at the same night.

^4^ Estimated ratio of mean of specified age class and mean of reference age class at the same night.
