# Supplementary material for: Sensor-supported measurement of adaptability of dogs (Canis familiaris) to a shelter environment: Nocturnal activity and behavior
Source: PLoS One. 2023 Jun 15;18(6):e0286429. doi: 10.1371/journal.pone.0286429 (PMC10270336; doi:10.1371/journal.pone.0286429)
Supplement: S14 Table — Estimated parameter values (EP) and 95% confidence intervals (CI) of body shaking (RPM) when the dog was in sight of the camera and active, during the night (0:00–4:00 h) for night (after intake) and other factors that significantly explained body shaking variability. Conditional F-testing revealed F, DF’s and significance of factors in the model. 1 Estimated mean in reference night, weight class and age class. 2 Estimated ratio of mean of specified night and mean on reference night. 3 Estimated ratio of mean of specified weight class and mean in reference weight class. 4 Estimated ratio of mean of specified kennel history and mean of reference kennel history. (DOCX) [file pone.0286429.s014.docx]

**S14 Table.** **Model results for nocturnal behavioural indicators of stress: Rate per minute (RPM) of body shaking in the shelter dog group.**

|  | | *RPM body shaking* | | | | | |
| --- | --- | --- | --- | --- | --- | --- | --- |
| **Category** | | Estimated | | Conditional F-test | | | |
|  |  | **EP** | **95% CI** | **F** | **NumDF** | **DenDF** | **Sign.** |
| Reference | Night 1, <10 kg, had kennel history | 0.05^1^ | 0.02-0.11 | 699.04 | 1 | 212 | <.0001 |
| Night | Night 2 versus night 1 | 1.64^2^ | 1.11-2.44 | 6.41 | 6 | 212 | <.0001 |
|  | Night 3 versus night 1 | 1.72^2^ | 1.14-2.60 |  |  |  |  |
|  | Night 5 versus night 1 | 2.80^2^ | 1.84-4.26 |  |  |  |  |
|  | Night 7 versus night 1 | 2.34^2^ | 1.51-3.62 |  |  |  |  |
|  | Night 9 versus night 1 | 2.65^2^ | 1.71-4.10 |  |  |  |  |
|  | Night 12 versus night 1 | 2.69^2^ | 1.72-4.21 |  |  |  |  |
| Weight class | 10-20 kg versus <10 kg | 0.86^3^ | 0.47-1.56 | 3.49 | 3 | 31 | 0.0273 |
|  | >20-30 kg versus <10 kg | 3.09^3^ | 1.60-5.95 |  |  |  |  |
|  | >30 kg versus <10 kg | 0.88^3^ | 0.45-1.72 |  |  |  |  |
| Kennel history | No history versus had history | 0.17^4^ | 0.08-0.39 | 10.84 | 2 | 31 | 0.0003 |
|  | Unknown versus had history | 0.54^4^ | 0.27-1.09 |  |  |  |  |

Estimated parameter values (EP) and 95% confidence intervals (CI) of *body shaking (RPM)* when the dog was in sight of the camera and active, during the night (0:00-4:00 h) for night (after intake) and other factors that significantly explained *body shaking* variability. Conditional F-testing revealed F, DF’s and significance of factors in the model.

^1^ Estimated mean in reference night, weight class and age class.

^2^ Estimated ratio of mean of specified night and mean on reference night.

^3^ Estimated ratio of mean of specified weight class and mean in reference weight class.

^4^ Estimated ratio of mean of specified kennel history and mean of reference kennel history.
