# Supplementary material for: Sensor-supported measurement of adaptability of dogs (Canis familiaris) to a shelter environment: Nocturnal activity and behavior
Source: PLoS One. 2023 Jun 15;18(6):e0286429. doi: 10.1371/journal.pone.0286429 (PMC10270336; doi:10.1371/journal.pone.0286429)
Supplement: Attachment — Submitted filename: Response to Reviewers.docx [file pone.0286429.s015.docx]

**Response to Reviewers**

Points raised by the academic editor:

1. Please ensure that your manuscript meets PLOS ONE's style requirements, including those for file naming. The PLOS ONE style templates can be found at 
<https://journals.plos.org/plosone/s/file?id=wjVg/PLOSOne_formatting_sample_main_body.pdf> and 
<https://journals.plos.org/plosone/s/file?id=ba62/PLOSOne_formatting_sample_title_authors_affiliations.pdf>

We have checked whether our manuscript meets the style requirements according to the templates and submission guidelines.

2.  You indicated that ethical approval was not necessary for your study. We understand that the framework for ethical oversight requirements for studies of this type may differ depending on the setting and we would appreciate some further clarification regarding your research. Could you please provide further details on why your study is exempt from the need for approval and confirmation from your institutional review board or research ethics committee (e.g., in the form of a letter or email correspondence) that ethics review was not necessary for this study? Please include a copy of the correspondence as an ""Other"" file.

Correspondence with the Animal Welfare Body of Utrecht is submitted as an “other” file, named “Copy correspondence AWB”.

3. We note that you have stated that you will provide repository information for your data at acceptance. Should your manuscript be accepted for publication, we will hold it until you provide the relevant accession numbers or DOIs necessary to access your data. If you wish to make changes to your Data Availability statement, please describe these changes in your cover letter and we will update your Data Availability statement to reflect the information you provide.

The full data file has now been added to OSF: DOI 10.17605/ [OSF.IO/J5XK2](http://osf.io/J5XK2) , and made publicly available.

4. Please review your reference list to ensure that it is complete and correct. If you have cited papers that have been retracted, please include the rationale for doing so in the manuscript text, or remove these references and replace them with relevant current references. Any changes to the reference list should be mentioned in the rebuttal letter that accompanies your revised manuscript. If you need to cite a retracted article, indicate the article’s retracted status in the References list and also include a citation and full reference for the retraction notice.

We have reviewed the reference list and made some (style) changes, please see the revised manuscript with Track Changes. No references were removed or added.

Points raised by the reviewer(s):

Reviewer #1: Thanks for submitting this paper which is a good additional to the literature on the effect of shelters of canine welfare. The paper is comprehensive and all methods and results fully presented. The conclusions seem to be reasonable.
DETAILED Comments
There are a number of English errors that will need to be corrected, e.g. Line 59 (may be), 108 (returned to their owners), the use of the word 'epoch' is unusual, 650 (admitted), 658 (indicating), 675 (safe), 705 (better balanced diet), 727 (data were) and 750 (dampen). Thank you for pointing out these errors. We have corrected them, explained what we meant with ‘epoch’.

I would like to see a better explanation why some results were excluded (e.g. 286-7) These data were excluded as these behaviours were observed too little to perform (mixed model) analysis. We chose for the 10% cutoff conform Hiby et al. (2006) and Part et al. (2014).
Line 339 - is this supposed to be after 13 days? Yes, thank you, corrected.
Line 733 - please explain what you mean by socialised less as used here. We changed the sentence into: One possible explanation is that smaller breeds are trained less [64] and have less socialisation experiences during puppyhood (7-16 weeks old) [65].
Line 765 - the word 'before' is repeated. We corrected this error.
